# Cancer dynamics for identical twin brothers

**DOI:** 10.1186/1742-4682-9-4

**Published:** 2012-02-06

**Authors:** Ali Ghaffari, Mostafa Khazaee

**Affiliations:** 1 Department of Mechanical Engineering, K.N. Toosi University of Technology, Pardis Street, Vanak Square, P.O. Box 19395-1999, Tehran, Iran; 2 Mechanical Engineering Department, Iran University of Science and Technology, Narmak, Tehran, 16846, Iran

## Abstract

In this paper, a new mathematical model is developed to represent the interaction between healthy and cancer cells in the human body, focusing on the role of environmental factors and quality of life in the progression of cancer. We have investigated the dynamic effects of inputs on cancer growth, and provide an explanation of how cancer has variable behavior patterns throughout the lives of different patients. The behavior of the system with input and its trajectory patterns are investigated using trajectory patterns and stability analysis. The analysis suggests that a proper treatment method should change the dynamics of the cancer instead of only reducing the population of cancer cells and treatment burden.

## Background

Some of the existing studies on cancer therapy are based on the assumption that cancer growth is a time invariant dynamic system [1]. They have been focused on the following objectives:

(i) To reduce and control the tumor mass such that a specified volume is obtained at the end of the treatment [2].

(ii) To lessen the treatment burden of patients. This method considers some constraints on the treatment policy [3].

(iii) To evaluate the number of injected cells that affect the equilibrium points of the immune system and thus may ultimately be dangerous [4].

Previous studies have investigated the effects of therapeutic inputs, which are considered to have direct effects on the system states [5]. However, the behavior of cancer changes as the disease progresses [6]. External stresses that represent destructive inputs, such as environmental and quality of life factors, can cause disability in the DNA repair genes [7]. They can also interfere with, and alter, the functions of regulatory growth signals (TGF-α), growth-inhibiting signals (TGF-β), and apoptosis (TP53) [6].

We are interested in analyzing how inputs alter the dynamics of the human body, and whether this is the main factor involved in the occurrence of cancer. This paper deals with a comparison between the responses of human body cells in a set of twin identical brothers who live under two different conditions, or inputs. In the next section, a modified model for the human body cells is presented. In the third section, the equilibrium points of the system and its linear approximation are calculated. The fourth section investigates the stability analysis of the system, using stability theorems and trajectory patterns. The fifth and final section discusses the results and summarizes the conclusions.

### Model

We have considered a set of identical twin brothers who were born with the same genetic structures. It is assumed that, after the birth, they reside and grow up in two different locations, and under different environmental conditions. One of them, say ***A***, lives in a polluted environment and under external stresses, but brother ***B ***does not.

The presented population model originates from [8]. The modifications in this model are described here. The dynamic behavior of an organ of the body that is affected by the cancer is given by the following time-variant equations.

(1) $$\frac{d}{dt}\left[\begin{array}{c} x\\ y \end{array}\right]=\left[\begin{array}{c} f_{1}\left(x,y,u,t\right)\\ f_{2}\left(x,y,u,t\right) \end{array}\right]$$

Where:

(2) $$f_{1}=a_{1}x\left(1-\frac{x}{K_{1}}\right)-\left(d_{1}+c\right)x-b_{1}xy,$$

(3) $$f_{2}=a_{2}y\left(1-\frac{y}{K_{2}}\right)-d_{2}y+cx-\left(b_{2}x+g\right)y.$$

The auxiliary equation of the system is

(4) $$\frac{dc}{dt}=c_{1}u\left(1-\frac{c}{K_{3}}\right)$$

In Eq. 1, *x *and *y *are the healthy and cancerous cell concentrations, respectively. Because the state variables are physiologically possible, their values are nonnegative, i.e., *x *≥ 0 and *y *≥ 0. The coefficients *a*_1 _and *a*_2 _represent the growth rates of the healthy and cancer cells, respectively. The growth of both healthy tissue and tumor decelerates as the concentrations of both the healthy and tumor tissues approach the carrying capacities *K*_1 _and *K*_2_, respectively [9]. The effect of the immune system is to kill the mutated and cancer cells in proportional rates *d*_1 _and *d*_2_. The immune system agents force the cancer cells to suicide through apoptosis [10]. The coefficient *c *represents the proportion of healthy cells whose genome has been disordered by external stresses. These cells initiate the neoplastic transformation, and are added to the tumor cells [11].

The tumor competes with healthy tissue for resources, such as blood, nutrients, and space, so the organ "feels" the tumor [12]. Moreover, these same cancer cells compete with each other. The competition coefficients between different cells are *b*_1_, *b*_2_, and *g*.

The effects of the input on the dynamics of the system are introduced by the registration of input history in the system coefficients. The destructive inputs, in addition to increasing cell mutation, predispose the cancer cells to intensification of the growth rate, and reduce the death rate by conferring the ability to evade the intracellular controlling mechanism and the immune system.

Cancer progression represents a macro-evolutionary process where karyotype change or genome replacement plays the key dominant role [13]. In the present work, the transformation rate of healthy cells to cancer cells is assumed to be proportional to the input magnitude *u*. The biotransformation coefficient saturates at a definite limit *K*_3_, which is related to the biological limits of body organs and the accumulation of external effects. The other variant parameters have similar formulations, but with different values and rates. When the destructive inputs terminate, their effects remain in the body and may or may not be compensated by the therapeutic inputs or by the body recovery.

The parameter values are listed in Table 1. These values should not be used for clinical applications, and are merely a means to approximate the cancer dynamics in the numerical analysis [8]. It may be shown by implementation of the Lipschitz theorem that Eq. 1 has a unique solution. The stability of the system is analyzed at the specified time intervals when the parameter variation can be ignored owing to low rates.

**Table 1:** Parameter estimations

Parameter	Unit	Estimated value								Source
		
		Birth	Youth		Middle age		Old		After Old	
		
		*A, B*	*A*	*B*	*A*	*B*	*A*	*B*	*B*	
***a***_1_	*week*^-1^	5					3.5	5	4.9	Estimated

***a***_2_	*week*^-1^	0.12	3.816	1.152	6	1.872	6.7	1.934	3.531	[4]

***K**_1_*	*Cell*	80 × 1000								[4]

***K**_2_*	*Cell*	90 × 1000								Estimated

***d***_1_	*week*^-1^	0.1								Estimated

***d***_2_	*week*^-1^	2.5	1	2.26	0.05	2.09	0.03	1.94	1.167	Estimated

***C***	*week*^-1^	0.001	0.032	0.010	0.05	0.016	0.05	0.019	0.028	Estimated

***b***_1_	*Cell*^-1^	0.06	0.191	0.063	0.3	0.107	0.6	0.115	0.206	[9]
		
***b***_2_	*week*^-1^	0.4	0.320	0.381	0.2	0.342	0.11	0.307	0.272	[3]

***G***	*week*^-1^	0.002	0.064	0.002	0.1	0.005	0.1	0.007	0.016	Estimated

***c***_1_	*week*^-1^	0.001								[4]

***K***_3_	*week*^-1^	0.05								Estimated

Some important properties of the model are presented here, as described in the following two statements.

*Statement 1. Let *$\left[\begin{array}{c} x\left(t\right)\\ y\left(t\right) \end{array}\right]$*be the solution of Eq. 1. Then the nonnegative orthant is invariant, i.e.*, $\left[\begin{array}{c} x\left(t\right)\\ y\left(t\right) \end{array}\right]\geq 0$.

Proof:

i) Notice that input *u *has no direct effect on the variables *x *and *y*, it can be concluded that if $\left[\begin{array}{c} x\left(0\right)\\ y\left(0\right) \end{array}\right]=\left[\begin{array}{c} 0\\ 0 \end{array}\right]$, then $\left[\begin{array}{c} x\left(t\right)\\ y\left(t\right) \end{array}\right]=\left[\begin{array}{c} 0\\ 0 \end{array}\right]$ for all *t *≥ 0.

ii) If the solution of Eq.1 approaches the horizontal axis from nonnegative orthant then, ${\left(ẋ\right|}_{x=0}=0$, ${\left(ẏ\right|}_{x=0}=\left[a_{2}\left(1-\frac{y}{K_{2}}\right)-\left(d_{2}+g\right)\right]y\geq \;0$ and *y *does not decrease, so its value becomes negative.

iii) If the solution of Eq.1 approaches the vertical axis from nonnegative orthant then,${\left(ẏ\right|}_{y=0}=0$, ${\left(ẋ\right|}_{y=0}=\left[a_{1}\left(1-\frac{x}{K_{1}}\right)-\left(d_{1}+c\right)\right]x\geq 0$ and *x *does not decrease, so its value becomes negative.

Hence, there is no solution that exits the first orthant.   □

Statement 2. The sum of state variables in Eq. 1 is exponent convergent in the first orthant. The region of convergence is contained in

(5) $$A=\left\{\left(x,y\right)|0\leq x+y\leq {W_{1}}_{max}\right\}$$

Proof:

Let *W *= *x *+ *y*. Then

$$\begin{array}{c} W_{1}=Ẇ+rW=x\left[a_{1}+r-\left(\frac{a_{1}+r}{K_{1}}x+d_{1}+b_{1}y\right)\right]\\ +y\left[a_{2}+r-\left(\frac{a_{2}+r}{K_{2}}y+d_{2}+b_{2}x+g\right)\right] \end{array}$$

Also the gradient of *W*_1 _may be shown as;

$$\nabla W_{1}=\left[\begin{array}{c} a_{1}+r-\left(2\frac{a_{1}+r}{K_{1}}x+d_{1}+\left(b_{1}+b_{2}\right)y\right)\\ a_{2}+r-\left(2\frac{a_{2}+r}{K_{2}}y+d_{2}+g+\left(b_{1}+b_{2}\right)x\right) \end{array}\right]$$

*W*_1 _decreases when *x *and *y *converge to infinity, thus it has a maximum value.

$$\begin{array}{c} \left|H_{W_{1}}\right|=\left|\begin{array}{cc} -2\frac{a_{1}+r}{K_{1}} & -\left(b_{1}+b_{2}\right)\\ -\left(b_{1}+b_{2}\right) & -2\frac{a_{2}+r}{K_{2}} \end{array}\right|\\ =4\frac{\left(a_{1}+r\right)\left(a_{2}+r\right)}{K_{1}K_{2}}-{\left(b_{1}+b_{2}\right)}^{2}>0 \end{array}$$

If the chosen value *r *is large enough, the above inequality is valid over the whole domain of analysis. The first element of $H_{W_{1}}$ is negative, thus (*x_ext_, y_ext_*) is the single maximum of *W*_1 _and the maximum value is *W*_1*max *_= *W*_1_(*x_ext_, y_ext_*). After some simplifications and letting

(6) $$W_{2}=W_{1}+\frac{1}{r}{W_{1}}_{max}.$$

the following inequality is achieved:

(7) $${Ẇ}_{2}+rW_{2}\leq 0$$

It may be shown [14] that for Eq.7 we have;

(8) $$W_{2}\left(t\right)\leq W_{2}\left(0\right)e^{-rt}.$$

Finally, with respect to Eqs. 6 and 8, the exponent convergence region is .   □

### Equilibrium points

Four equilibrium points of Eq. 1 with *u *= 0 are calculated as:

(9) 1)x=0,y=0

(10) $$2)x=0,y=\frac{K_{2}\left(a_{2}-d_{2}-g\right)}{a_{2}}=-\frac{\beta_{2}}{\alpha_{2}}$$

(11) $$3)x=\alpha_{6}+\alpha_{7},y=\alpha_{1}\left(\alpha_{6}+\alpha_{7}\right)+\beta_{1}$$

(12) $$4)x=\alpha_{6}-\alpha_{7},y=\alpha_{1}\left(\alpha_{6}-\alpha_{7}\right)+\beta_{1}$$

Where:

$$\begin{array}{c} \alpha_{1}=-\frac{a_{1}}{K_{1}b_{1}},\;\beta_{1}=\frac{a_{1}-d_{1}-c}{b_{1}},\;\alpha_{2}=-\frac{a_{2}}{K_{2}},\;\beta_{2}=a_{2}-d_{2}-g,\;\alpha_{3}=\alpha_{1}\left(\alpha_{1}\alpha_{2}-b_{2}\right),\\ \alpha_{4}=2\alpha_{1}\alpha_{2}\beta_{1}-b_{2}\beta_{1}+\alpha_{1}\beta_{2}+c,\;\alpha_{5}=\beta_{1}\left(\alpha_{2}\beta_{1}+\beta_{2}\right),\;\alpha_{6}=\frac{-\alpha_{4}}{2\alpha_{3}},\;\alpha_{7}=\frac{\sqrt{{\alpha_{4}}^{2}-4\alpha_{3}\alpha_{5}}}{2\alpha_{3}}. \end{array}$$

The linear approximation of Eq.1 is given by

(13) $$\left[\begin{array}{c} {ẋ}^{*}\\ {ẏ}^{*} \end{array}\right]=AZ^{*}+B_{H.O.T.}+C$$

Where:

$$\begin{array}{c} A=\left[\begin{array}{cc} b_{1}\left(\beta_{1}-ȳ\right)-2\frac{a_{1}}{K_{1}}\bar{x} & -b_{1}\bar{x}\\ c-b_{2}ȳ & a_{2}-2\frac{a_{2}}{K_{2}}ȳ-d_{2}-g-b_{2}\bar{x} \end{array}\right],\\ B_{H.O.T.}=-\left[\begin{array}{c} \frac{a_{1}}{K_{1}}{x^{*}}^{2}+b_{1}x^{*}y^{*}\\ \frac{a_{2}}{K_{2}}{y^{*}}^{2}+b_{2}x^{*}y^{*} \end{array}\right],\;C=\left[\begin{array}{c} \left(a_{1}-d_{1}-c\right)\bar{x}-\frac{a_{1}}{K_{1}}{\bar{x}}^{2}-b_{1}\bar{x}ȳ\\ \left(a_{2}-d_{2}-g\right)ȳ-\frac{a_{2}}{K_{2}}{ȳ}^{2}-b_{2}\bar{x}ȳ+c\bar{x} \end{array}\right] \end{array}$$

The higher order terms are neglected around the origin (x*, y*) = (0, 0) and the last term *C *is equal to zero at the equilibrium points.

### Stability analysis

We study the stability of the equilibrium points of Eq. 1 in this section. The results of the analysis are stated as follows;

*i. If the equilibrium point 3 is located in the first orthant and the equilibrium point 2 is not, then the state variables of Eq. 1 will converge to equilibrium point 3 (the healthy state)*.

*ii. If the equilibrium points 2 and 3 are located in the first orthant, then the state variables of Eq. 1 will converge to one of these two points*.

*iii. If the equilibrium point 2 is located in the first orthant and the equilibrium point 3 is not, then the state variables of Eq. 1 will converge to the equilibrium point 2 (the cancer state)*.

Proof:

1) At the equilibrium point 1, *x *= 0, y = 0, the eigenvalues of *A *are;

$$\left\{\begin{array}{c} \lambda_{1}=a_{1}-\left(d_{1}+c\right)\;\\ \lambda_{2}=a_{2}-\left(d_{2}+g\right) \end{array}\right)$$

As shown in Table 1, the values of *a*_1 _are larger than the sum of *d*_1 _and *c*. Also *a*_2 _is larger than the sum of *d*_2 _and *g*. Thus the eigenvalues of equilibrium point 1 are always positive and the origin is an unstable node.

2) At the equilibrium point 2, $x=0,y=-\frac{\beta_{2}}{\alpha_{2}}$, the eigenvalues of *A *are;

$$\left\{\begin{array}{c} \lambda_{1}=b_{1}\left(\beta_{1}+\frac{\beta_{2}}{\alpha_{2}}\right)\text{,}\\ \lambda_{2}=-\left(a_{2}-d_{2}-g\right) \end{array}\right)$$

In Eq.10 *y *is positive, then *β*_2_/*α*_2 _< 0. Also, we notice from Table 1 that if the value of *β*_2_/*α_2 _*is larger than *β*_1_, then the equilibrium point 2, if it exists, is a stable node.

3) At the equilibrium point 3, *x *= *α*_6 _+ *α*_7_, *y *= *α*(*α*_6 _+ *α*_7_) + *β*_1_.

Noticing that *x *and *y *in Eq. 11 are in the first orthant, then *α*_6 _+ *α*_7 _> 0 and *α*_1_(*α*_6 _+ *α*_7_) + *β*_1 _> 0. The principal minors of -*A *at this equilibrium point are as follows:

$$\begin{array}{c} {\text{Δ}}_{1}=\frac{a_{1}}{K_{1}}\left(\alpha_{6}+\alpha_{7}\right)>0,\\ {\text{Δ}}_{2}=\{2\frac{{a_{1}}^{2}a_{2}}{K_{1}K_{2}b_{1}}\left[1-\frac{1}{K_{1}}\left(\alpha_{6}+\alpha_{7}\right)\right]\\ +\frac{a_{1}}{K_{1}}\left(2b_{2}\left(\alpha_{6}+\alpha_{7}\right)+d_{2}+g-2\frac{a_{2}\left(d_{1}+c\right)}{K_{2}b_{1}}-a_{2}\right)\\ +b_{1}\left(c-b_{2}\frac{a_{1}-d_{1}-c}{b_{1}}\right)\}\left(\alpha_{6}+\alpha_{7}\right)>0 \end{array}$$

This means that the coefficient matrix *A *is negative definite at this equilibrium point and it is a stable node.

4) At the equilibrium point 4, *x *= *α*_6 _- *α*_7_, y = *α*_1_(*α*_6 _- *α*_6_) + *β*_1_.

Noticing that *x *and *y *in Eq.12 are in the first orthant, then *α*_6 _- *α*_7 _> 0 and *α*_1_(*α*_6 _- *α*_7_) + *β*_1 _> 0. The principal minors of -*A *at this equilibrium point are as follows:

$$\begin{array}{c} {\text{Δ}}_{1}=\frac{a_{1}}{K_{1}}\left(\alpha_{6}-\alpha_{7}\right)>0,\\ {\text{Δ}}_{2}=\{2\frac{{a_{1}}^{2}a_{2}}{K_{1}K_{2}b_{1}}\left[1-\frac{1}{K_{1}}\left(\alpha_{6}-\alpha_{7}\right)\right]\\ +\frac{a_{1}}{K_{1}}\left(2b_{2}\left(\alpha_{6}-\alpha_{7}\right)+d_{2}+g-2\frac{a_{2}\left(d_{1}+c\right)}{K_{2}b_{1}}-a_{2}\right),\\ +b_{1}\left(c-b_{2}\frac{a_{1}-d_{1}-c}{b_{1}}\right)\}\left(\alpha_{6}-\alpha_{7}\right)<0 \end{array}$$

Therefore, if this equilibrium point exists, then it is an unstable saddle point.   □

### Numerical analysis

In this section, the trajectory patterns of the dynamic systems for the twin brothers are evaluated. The life spans of both brothers are divided into three stages and the dynamic behaviors of the systems are analyzed before and after the presence of inputs. The first stage is from the birth to 850 weeks, the second stage is from 850 to 1950 weeks, and the third stage is from 1950 weeks to 2900 weeks.

The following figures illustrate the trajectory patterns of the dynamic systems representing twin brothers ***A ***and ***B ***at the beginning of the three stages of their life.

**Figure 1:**
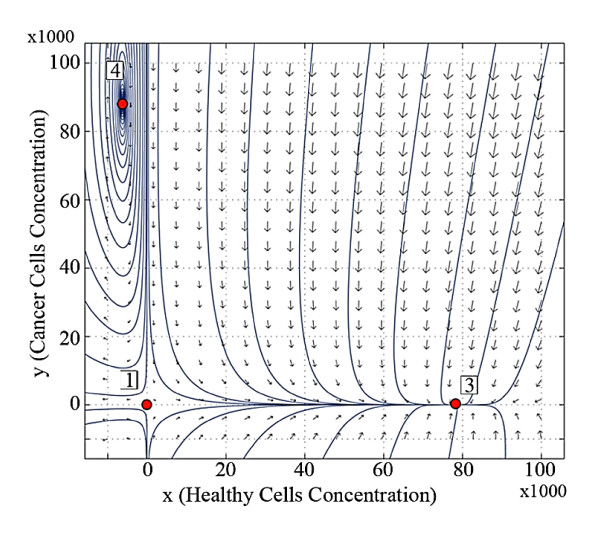
**Trajectory pattern at the beginning of the first stage of life (at birth) for both brothers *A *and *B***.

Figure 1 shows the trajectory patterns at the beginning of the first stage of life (at birth) for both brothers ***A ***and ***B***. It shows that the equilibrium points 2 and 4 are not located in the positive orthant. Therefore, they are not feasible in any organs of the newborn bodies. This figure also indicates that the only stable equilibrium point of the system is point 3 and the equilibrium point 1 is unstable. The system converges to the equilibrium point 3, if the initial values are in the positive orthant for two newborn brothers.

**Figure 2:**
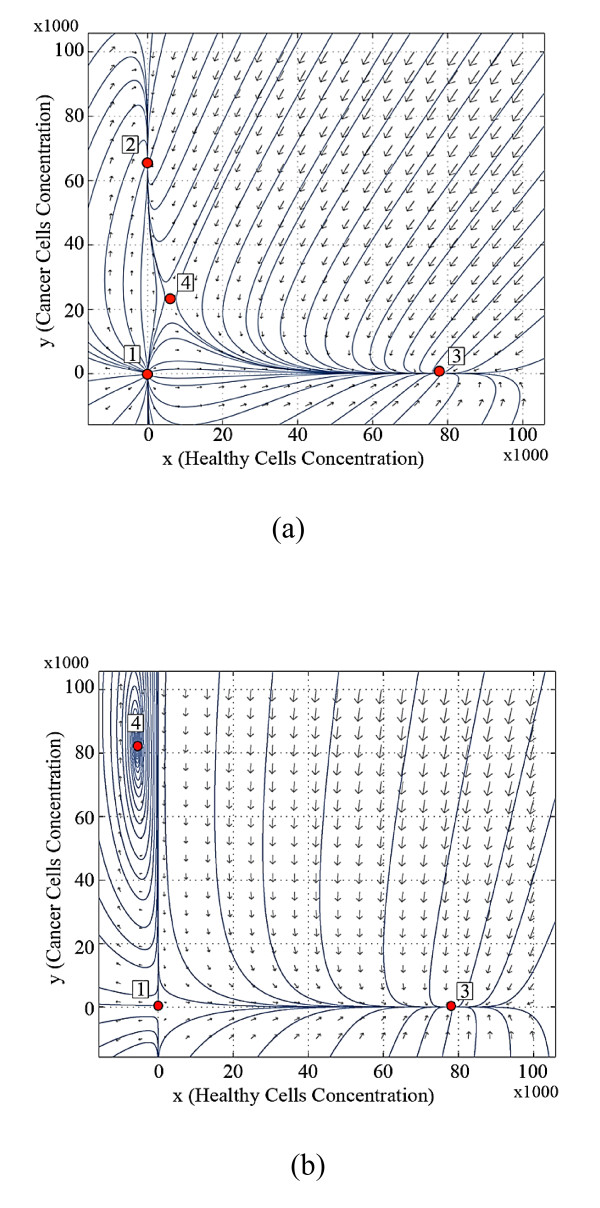
**Trajectory patterns at the beginning of the second stage of life, (a) for brother *A *(affected by cancer) and (b) for brother *B *(healthy brother)**.

Figure 2 shows the trajectory pattern at the beginning of the second stage of life for the twin brothers, where brother ***A ***has been under the effect of destructive inputs during the first stage of life, but brother ***B ***has not.

Figure 2-a shows that for brother ***A***, the equilibrium points 2 and 4 are brought to the positive orthant by receiving the destructive input for a limited time, and the trajectories converge to the healthy equilibrium point 3 from a large portion of the state space. However, the behavior of the system for brother ***B***, as shown in Figure 2-b, is similar to Figure 1, with the positions of the equilibrium points 2 and 4 showing only minor changes.

**Figure 3:**
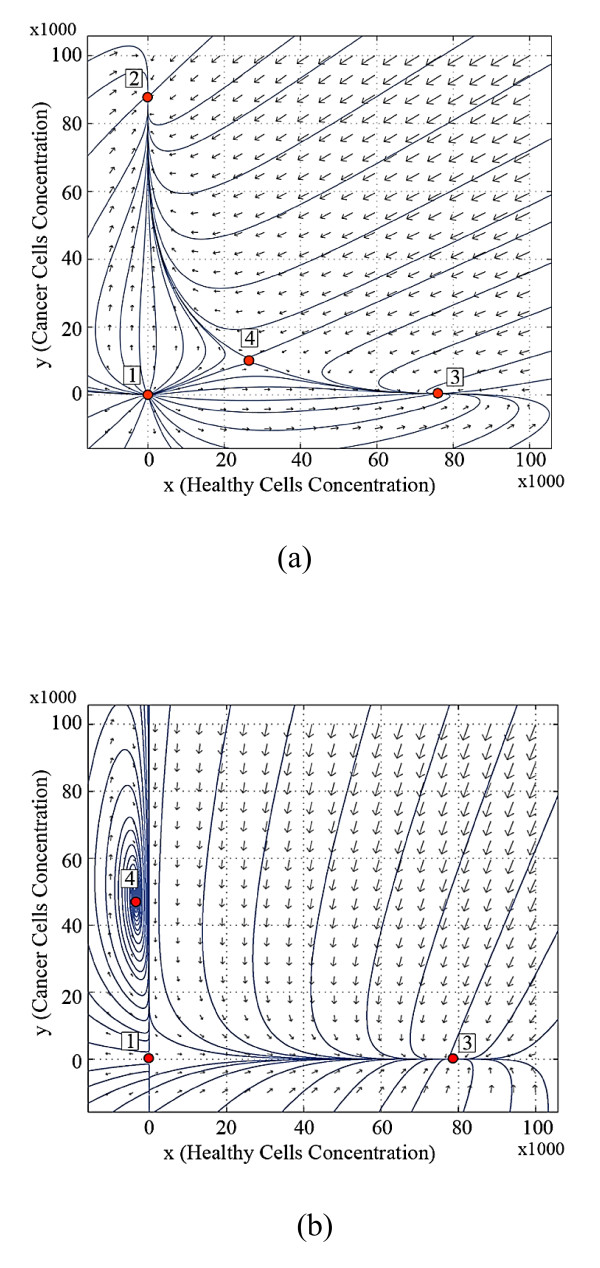
**Trajectory patterns at the beginning of the third stage of life, (a) for brother *A *(affected by cancer) and (b) for brother *B *(healthy brother)**.

Figure 3 shows the trajectory pattern at the beginning of the third stage of life for the twin brothers, where brother ***A ***has been under the effect of destructive inputs during the second stage of life, but brother ***B ***has not. Figure 3-a indicates that the attraction area of equilibrium point 2 is larger than 3 for brother ***A***. The position of equilibrium point 3 remains approximately constant, but the equilibrium points 2 and 4 change as ***A ***becomes older and encounters more destructive inputs. These changes lead to an increase in the probability of the incidence of cancer. The destructive input affects the system dynamics and the effects are accumulated in the body of ***A***. The input is assumed to end, but the behavior of the system is changed.

The body organ of ***B ***is changed a little, but the system does not pose any unstable behavior in the first orthant, as is seen in Figure 3-b. The small variation is due to aging and the probable effect of limited destructive inputs.

**Figure 4:**
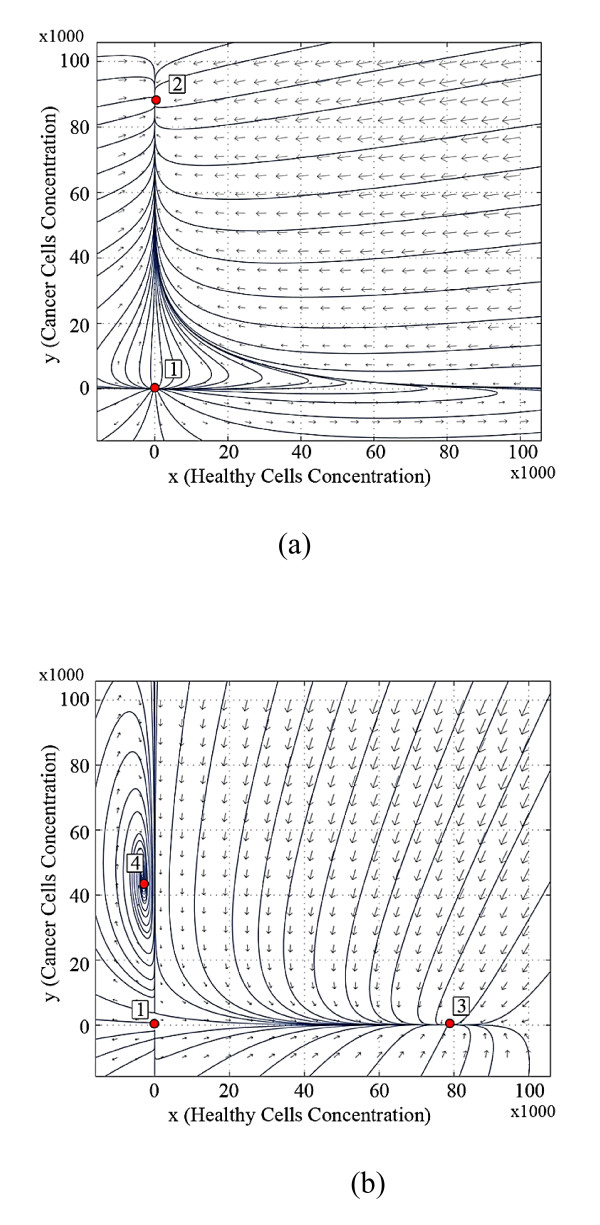
**Trajectory patterns at the end of the third stage of life, (a) for brother *A *(affected by cancer) and (b) for brother *B *(healthy brother)**.

Figure 4 shows the trajectory pattern at the end of the third stage of life for the twin brothers, where brother ***A ***has been under the effect of destructive inputs during the third stage of life, but brother ***B ***has not. The only stable equilibrium is equilibrium 2 which is located in the nonnegative orthant at the end of the third stage of life for brother ***A***, as is shown in Figure 4-a. This brother will die of cancer if the dynamics of the system is not modified. All trajectories in the positive orthant converge on to the cancer equilibrium point 2. Therefore, the cancer is not treated by minimizing the number of cancer cells. There is a low rate of dynamics change in the body of ***B***. The behavior of the system does not transform such that equilibrium point 2 is located in the nonnegative orthant and thus affects the health condition of ***B***. However the equilibrium points 2 and 4 near the positive orthant of state space. The health margin of ***B ***is not as it was at birth.

Finally, a situation is analyzed in which ***B ***(the healthy brother) encounters destructive inputs from 2900 to 3200 weeks.

**Figure 5:**
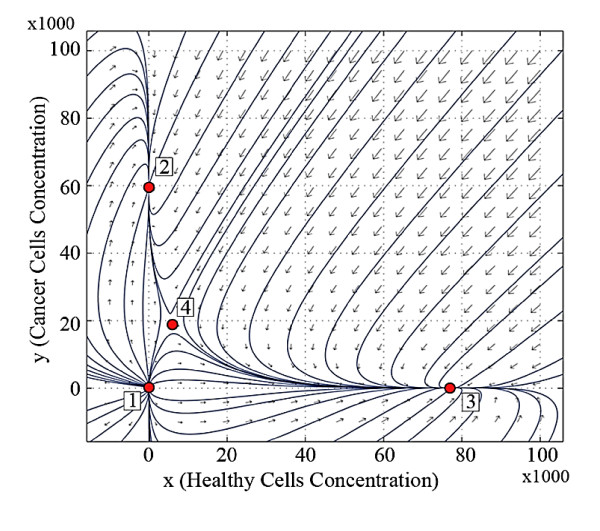
**Trajectory patterns at the 3200th week of of life, for brother B (healthy brother) after the presence of destructive inputs for some years**.

Figure 5 shows the trajectory patterns for brother ***B***, where he has been under the effect of destructive inputs from 2900 to 3200 weeks. It shows that the destructive inputs change the dynamics of the body organ such that the equilibrium points 2 and 4 enter the nonnegative orthant. The behavior of the system in this figure is similar to Figure 2-a for brother ***A***, but the dynamics change in Figure 2-a occurs over a period of 850 weeks, while Figure 5 shows the change within 300 weeks.

## Discussion and conclusion

The stable dynamics for the newborn brothers at birth means that they do not have cancer. If the equilibrium points of the system are in the nonnegative orthant then the system will settle in the equilibrium points 2 or 3. The history of the body organ's dynamics during the life of brother ***B ***indicates his healthy condition over his life (Figures 1, 2-b, 3-b, and 4-b). This brother is not afflicted by cancer since he has not faced the destructive inputs. The variation of the system dynamics where the destructive inputs are sufficiently effective, demonstrate cancer formation and its progress in the body of brother ***A ***(Figures 1, 2-a, 3-a, and 4-a). The stable behavior for ***A ***at birth is gradually converted to less stable conditions. The attraction domain of equilibrium point 2, indicating the cancer cells, becomes larger than equilibrium 3, indicating healthy cells. Thus, the cancer incidence is increased. In such a system, if the number of cancer cells reaches a specified limit, then the patient will die. In our model, brother ***A ***reaches the stage in which no treatment is able to cure the patient completely and he dies.

This research shows that focusing on the "indelible changes of the system dynamics" is the best way to describe the cancer formation process. The other major conclusions are as follows:

a) The changes in the dynamics of the system occur gradually in the body of the patient. Thus, the duration of effective destructive inputs is directly related to the probability of the cancer occurrence. This can be seen in Figures 2-a and 3-a.

b) As shown in Figures 2-b, 3-b, and 4-b, the behavior of the system for the healthy brother ***B***, who is not subjected to the destructive inputs, also changes as he becomes older.

c) The effects of environmental and life quality factors are the main causes for the onset of cancer, in bodies where there are no dominant hereditary genetic disorders.

d) The sensitivity of the old healthy brother ***B ***to the destructive inputs is greater than that of the young affected brother ***A***, and the system dynamics changes more rapidly for him.

Our next objective is to find a proper therapeutic input that can move equilibrium point 2 out of the nonnegative space. This treatment method would guarantee the impossibility of tumor recurrence. The undesirable changes of the system should be modified by using the corrective inputs, and the treatment not restricted to the system outputs. Furthermore, in future work, the parameters, especially the input, should relate to the qualitative conditions of a patient's life and to the clinical data.

## Competing interests

The authors declare that they have no competing interests.

## Authors' contributions

AG and MK participated in numerous discussions leading to the development of the idea, jointly wrote the manuscript and edited the final version of the paper. All authors read and approved the final manuscript.
